# From molecular chaperones to membrane motors: through the lens of a mass spectrometrist

**DOI:** 10.1042/BST20160395

**Published:** 2017-02-15

**Authors:** Carol V. Robinson

**Affiliations:** Department of Chemistry, University of Oxford, South Parks Road, Oxford OX1 3QY, U.K.

**Keywords:** ion mobility, mass spectrometry, membrane proteins, molecular chaperones

## Abstract

Twenty-five years ago, we obtained our first mass spectra of molecular chaperones in complex with protein ligands and entered a new field of gas-phase structural biology. It is perhaps now time to pause and reflect, and to ask how many of our initial structure predictions and models derived from mass spectrometry (MS) datasets were correct. With recent advances in structure determination, many of the most challenging complexes that we studied over the years have become tractable by other structural biology approaches enabling such comparisons to be made. Moreover, in the light of powerful new electron microscopy methods, what role is there now for MS? In considering these questions, I will give my personal view on progress and problems as well as my predictions for future directions.

## Introduction

In the early days of applying mass spectrometry (MS) to structural biology, our development of methods to study the folding of proteins was viewed with much cautious scepticism. ‘Life, as we know it, goes on in water’, how then could we study proteins in the vacuum of a mass spectrometer? [1] This opinion piece voiced a real concern shared by many. Over the intervening years, however, we and others have largely silenced the critics and established the field of gas-phase structural biology, primarily through unequivocal demonstration of subunit stoichiometry, and by elucidating the conformation of gas-phase assemblies by means of ion mobility (IM) MS. What follows, and prompted by this award, is a personal account of my experiences during this time. In writing my perspective, it is important to emphasise that I am indebted to the contributions of numerous scientists in the field, many of whom have become personal friends and are a constant source of inspiration.

## The early years

That the folded core of a protein could be maintained in the gas phase of a mass spectrometer, as evidenced by hydrogen–deuterium exchange protection, was for me the starting point of my interests in using MS in structural biology [2]. These early observations led me to try to capture protein folding in the presence of cofactors [3] and subsequently in the presence of the molecular chaperone GroEL [4]. That the folded core of these proteins responded differently when with or without cofactors and chaperones implied that the gas phase could faithfully reproduce solution-phase folding events. Much remained to be explored about the extent of folded structure that survives the phase change however, and the time scales involved in transfer into the gas phase, but for me this was evidence that at least some aspects of protein secondary structure could be preserved.

It was during these protein-folding experiments that I first noticed that the appearance of folded protein coincided with the time point at which binding of the cofactor took place. This implied that cofactor binding was not the result of non-specific interactions, but required the correctly folded protein [5]. This was a turning point and started my long and continued interest in non-covalent interactions and their representation in the gas phase of the mass spectrometer [6].

This research field was more crowded than the protein-folding arena since it has obvious links to the pharmaceutical industry [7], and is the subject of many patents and proposals to use ligand binding deduced by MS for high-throughput screening [8]. However, there were initial difficulties with the nature of the interactions between proteins and their cognate ligands, with hydrophobic interactions considered less favourable for survival in the gas phase than ionic ones [5,9]. Moreover, variability in the experimental set-up and in control of the electrospray process made it difficult to compare results across different MS platforms. It became apparent, however, that the initial aqueous droplet size in the early stages of electrospray was critical to ameliorate the otherwise harsh desolvation conditions necessary to release proteins from solution, and that ‘soft’ gas-phase collisions are required to absorb translational and vibrational energy. In understanding these factors, the use of aqueous buffered solutions and elevated pressure regimes was found to enhance the survival of protein complexes in the gas phase [10].

Having started my research studying the influence of molecular chaperones on folding by releasing protein substrates from the GroEL_14-mer,_ the next obvious step for me was to try to retain the large GroEL complex intact in the gas phase of the mass spectrometer. Using a time-of-flight mass spectrometer, we recorded our first mass spectra of GroEL by employing nanoflow capillaries and elevated pressures [11]. Our first mass spectrum demonstrated the 14-mer stoichiometry of GroEL, and hinted at the overall packing of subunits within the complex through the preservation of non-covalent interactions in heptameric assemblies. Subsequently, we designed a high-mass quadrupole time-of-flight mass spectrometer for transmission and selection of discrete mass to charge regions of the mass spectrum of protein complexes [12,13]. Using this instrumentation, we showed not only that we could preserve the 800-kDa GroEL complex but also that we could perform tandem MS.

## Exploiting heterogeneity

Studying molecular chaperone assemblies, comprising one protein subunit, prompted us to extend our approach to study heteromeric complexes. Introducing mixtures of dodecameric small heat shock proteins (sHSP) from pea (PsHSP18.1) and from wheat (TaHSP16.9), allowing them to equilibrate, and following the kinetics of this reaction by MS, we could show that exchange proceeds via sequential incorporation of subunits, with dimeric species being the principal units of exchange. We concluded that the sHSP complexes are in a rapidly exchanging dissociation/association equilibrium with sub-oligomeric forms and were able to obtain rate constants for the exchange of subunits between species [14].

Along a similar line, we demonstrated that we could monitor assembly pathways of heteromeric chaperones from component subunits by incubating two different subunits (α and β) of the MtGimC complex [15]. This was carried out in advance of the X-ray crystal structure of MtGimC, and our results correctly predicted the hexamer with two α subunits forming the core surrounded by four peripheral β subunits. We also measured the thermal stability of this complex by constructing a heated nanoflow device to enable us to measure thermal stability *in situ* [16]. This offered a new capability since heat-induced changes of individual proteins are readily monitored by many spectroscopic methods, but changes in non-covalent complexes of biomolecules are more challenging to interpret. Applying this technique to the study of the 200-kDa complex of TaHSP16.9 revealed both its dissociation into sub-oligomeric species and an increase in its size and polydispersity at elevated temperatures.

So began an interesting decade of uncovering the polydispersity of molecular chaperones [17], of measuring subunit exchange dynamics, and of extracting rate constants for their associations [18]. It was also tempting during this time to expand capabilities to ever-larger complexes, including those containing DNA/RNA. Although, in general, the presence of nucleic acids complicates mass spectra, with their propensity to bind metal ions and tendency to heterogeneity, when decorated with protein, reasonable resolution can be obtained. Early forays into virus capsids [19], which have since been surpassed by others in the field with spectacular effect [20,21], demonstrated the feasibility of these approaches and opened new avenues of research into viral architecture [22].

Ribosomes, which are protein–RNA complexes, represented a considerable challenge not least because of the heterogeneity of initial preparations from *Escherichia coli* [23]. Ribosomes from *Thermus thermophilus* yielded much greater mass spectral resolution, perhaps correlated to their greater stability and homogeneity. Mass spectra also highlighted the unexpected heptameric stoichiometry of the stalk complex [24], shown first by MS, and confirmed crystallographically with truncated forms of L10 and the N-terminal domain of L12 [25].

Exploiting the ability of MS to probe dynamic exchange reactions, we were also able to link acetylated forms of the L12 protein (L7) with enhanced interactions with the ribosome [26] and used MS to investigate the stoichiometry of ribosomal stalk complexes from bacteria, eukaryotes, and archaea *in situ* on the ribosome. Specifically, we targeted ribosomes from organisms with different optimal growth temperatures. Our results showed that, for the mesophilic bacterial ribosomes, the stalk complexes are exclusively pentameric, and entirely heptameric in the case of thermophilic bacteria. Only pentameric stalk complexes were observed in eukaryotic species. We also were surprised to find that, for mesophilic archaea, both pentameric and heptameric stoichiometries are present simultaneously within a population of ribosomes. Moreover, the ratio of pentameric-to-heptameric stalk complexes changed during the course of cell growth [27]. These results were later supported by computational analysis and validated by quantitative MS [28].

The importance of dimeric interactions in the stalk complex was demonstrated by the exchange of subunits within the stalk complexes, while they were attached to ribosomes in different functional states [29]. By incubating isotopically labelled intact ribosomes with their unlabelled counterparts, we monitored the exchange of the labile stalk proteins by recording mass spectra as a function of time. We proposed a mechanism whereby exchange proceeds via L7/L12 monomers and dimers. We also compared exchange of L7/L12 from free ribosomes with exchange from ribosomes in complex with elongation factor G (EF-G), trapped in the post-translocational state by fusidic acid. The binding of EF-G does not modify interactions between the L7/L12 monomers but rather one of the four monomers, and as a result one of the two dimers becomes anchored to the ribosome–EF-G complex preventing their free exchange [29].

The yeast RNA exosome stands out as an early success in our quest to define the structural organisation of subunits using MS restraints alone. Through solution disruption measurements, it became possible to generate overlapping sub-complexes and to obtain pairwise interactions that form the ring structure [30]. The model proposed using MS was subsequently validated by X-ray crystallography [31]. This encouraged us to go further, generating interaction maps of both yeast and human eIF3 complexes. For the 13 subunits of the human eIF3 complex, we generated multiple sub-complexes in solution and assembled them into a model constrained by low-resolution EM density [32]. We can now compare our data for the 13-subunit model to the structure of the core complex for the 43S pre-initiation complex (8 subunits) determined last year using cryo-EM. The four ternary complexes that we deduced are entirely consistent with this structure, and the linear and compact arrangements defined by IM (see below) are in agreement with the structure shown for the core complex [33].

While in all cases the stoichiometry has been found to be correct, in some cases our models have not turned out to be entirely accurate predictions. It is interesting to consider how this arises. In the decameric structure of eIF2B [34], the dimeric nature of the two pentamers meant that it was difficult to assign complexes unambiguously since there were two copies of all subunits, unlike the situation for eIF3 or the yeast exosome where all subunits are unique and therefore can be distinguished by mass. Even with the aid of chemical cross-linking, it was not possible to determine a unique solution for eIF2B without invoking homology modelling. The high-resolution X-ray crystal structure of eIF2B revealed a different arrangement of protein subunits in the core, but confirmed the unexpected decameric stoichiometry of the complex.

## Adding a new dimension

It was not until 2005 that we began to look at the shape of protein complexes in the gas phase using IM-MS, a technique that had previously been pioneered for the study of small molecules and individual proteins. Applying these methods to protein complexes was fascinating and led to new insights into the shape and multiple conformations exhibited by proteins in solution and preserved in the gas phase. With this extra dimension, we explored packing of a range of protein complexes in different topological states [35] and compared these data with dissociation data in solution [36].

While, for most of our experiments, we were keen to maintain proteins in their compact folded states, we also become interested in unfolding proteins as a means of investigating their stability. We began these experiments by carrying out protein unfolding of transthyretin (TTR). Many different states were populated along the unfolding pathway and it was unclear how to assign these intermediates [37]. We did demonstrate, however, that wild-type TTR was able to resist unfolding more readily when bound to its natural ligand thyroxine than to the disease-associated variants, implying that this approach would be viable for identifying ligands that stabilise protein complexes in the gas phase [38].

We used IM to distinguish different locations within the EM density for eIF3 f,h,m, which was a close packed trimer with all subunits in contact, from eIF3 e,l,k, which has a linear arrangement with subunit l in the centre [35]. The overall conformation of these two trimers is in agreement with the structure of the core complex [33]. This correspondence is not always the case however, particularly when flexible hinge regions are present in protein assemblies. For example, in antibodies or in the case of p53 [39] wherein intrinsically disordered regions of the protein collapse on transition from the solution to gas phase, these yield smaller than theoretically calculated collision cross sections.

## Collecting and validating

Ever since the first spectra of GroEL, it became a personal goal to attempt to soft-land protein complexes within the mass spectrometer and to use EM to visualise these molecules. By building a retractable probe incorporating a versatile target holder, and modifying the ion optics of a commercial mass spectrometer, we were able to show that we can steer the macromolecular ion beam onto a target for imaging by means of transmission electron microscopy and atomic force microscopy. Our data for the tetradecameric chaperonin GroEL showed that not only are the molecular volumes of the landed particles consistent with the overall dimensions of the complex, but also that their gross topological features can be maintained [40]. We extended this approach to a second cage-like protein complex, ferritin. Following negative staining, images of the soft-landed complexes revealed that their structural integrity is largely conserved, with the characteristic central cavity of apoferritin and iron core of holoferritin, surviving the phase transition from liquid to gas, soft-landing, and dehydration in vacuum [41].

## Membrane proteins — the final frontier?

Perhaps for me my ultimate goal has been to study membrane proteins from an environment that is as close to the natural membrane as possible. Interestingly, the commentary back in 1995 [1] said that ‘The vacuum being very apolar can be thought of as a very hydrophobic medium, much like the lipid of a biomembrane’, suggesting that membrane proteins and MS may be very compatible with each other. Despite the obvious advantage of using the mass spectrometer to replicate the membrane environment, it was not until more than a decade after analysing our first soluble protein complexes that we succeeded in transporting an intact membrane protein complex into the gas phase of the mass spectrometer [42]. In 2008, we managed to maintain the heterotetrameric ABC transporter BtuCD in the gas phase and to observe both nucleotide and lipid binding [43]. This was the start of a whole new era — one in which we could start to explore the complexities of membrane proteins and their myriad of interactions with lipids.

One of the first membrane protein complexes I was keen to tackle was that of the rotary ATPases. These fascinating proteins were at that time recalcitrant to most structural biology approaches in their intact state. They were therefore of considerable interest as we reasoned that if we could maintain them intact in the mass spectrometer, we would be able to learn something about the communication between the soluble head and the membrane-embedded base. From our first mass spectra, we were able to define the precise subunit stoichiometry and the lipid cohort, and even an unexpected nucleotide-binding site [44]. Using IM-MS, we compared the conformational dynamics of the intact ATPase from *T. thermophilus* with those of its membrane and soluble sub-complexes. We defined regions with enhanced flexibility and assigned these to distinct subunits within the overall assembly. By isolating complexes at different phases of cell growth and manipulating nucleotides, metal ions, and pH during isolation, we were able to show differences that could be related to conformational changes in the Vo complex triggered by ATP binding. Subsequently, we went on to show how changes in the phosphorylation status in the soluble F_1_ subunit of the chloroplast ATPase affect nucleotide-binding sites [45]. To do this, we developed a comparative cross-linking strategy [46] to differentially label phosphorylated and dephosphorylated forms and to show how nucleotide binding was linked to phosphorylation status. Comparing these MS studies with recent cryo-EM structures for rotary ATPases reveals that not only were the subunit stoichiometries determined by MS correct, but also that the conformational heterogeneity of the membrane-embedded regions, revealed by IM-MS, also featured in the structures deduced by cryo-EM [47].

Returning to the theme of ABC transporters, given that our first success was with an ABC transporter BtuCD, we turned our attention to P-glycoprotein (P-gp) due to its importance in drug resistance mechanisms. We probed the synergy between lipid and drug binding by monitoring conformational changes within the pump [48]. This was at first considered controversial given that the X-ray crystal structures of most ABC transporters at that time were in either inward- or outward-facing conformations dependent on the binding of nucleotides [49]. Recent EM studies have, however, revealed a broad spectrum of conformations for the ABC transporters MsbA and P-gp [50], consistent with the dynamic equilibrium of states proposed from our IM-MS data. Our mass spectra of TmrAB, an ABC transporter from *T. thermophilus*, revealed lipids that were critical for structure and function [51] and were also clearly visible in high-resolution EM data, although in this case assigned to the detergent micelle [52].

**Figure 1.**
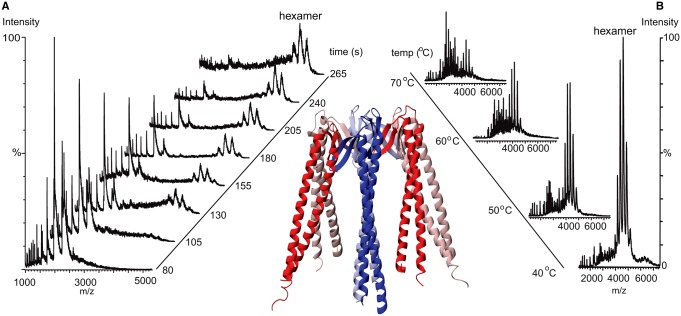
(**A**) The assembly of MtGimC from its component subunits. α-Subunits (red) form a dimeric platform for assembly of the four β-subunits monitored in real time by MS. (**B**) The thermal stability of the MtGimC hexamer investigated as a function of temperature. Reproduced with permission from ref. [15]. *Inset*, X-ray structure of the MtGimC complex [62] from the co-ordinates 1FXK.

**Figure 2.**
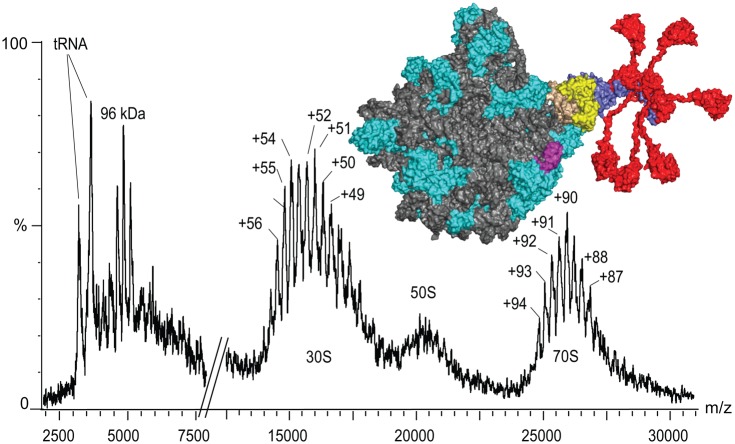
The mass spectrum of intact ribosomes from *T. thermophilus* at a mass of 2.3 MDa, and with >50 different proteins and 3 large RNA molecules with masses of 2 325 463 ± 2000 Da and 798 165 ± 164 Da for the 70S and 30S, respectively. The complex at 96 kDa is consistent with a heptameric stalk as opposed to the canonical pentameric stalks observed in all prokaryotic ribosomes up until this point. Subsequent X-ray analysis of L10 and its interactions with L12 confirmed this unexpected stoichiometry and enabled modelling of the 50S subunit with a heptameric stalk [25].

Combining our membrane protein MS with the unfolding experiments described above, we were able to identify lipids that affect the structure of the ammonia channel (AmtB) and for the function of aquaporin Z. That led to the first lipid-bound structure of AmtB [53]. Similarly, MS was used to inform the optimal conditions for recovering the membrane pore protein lysenin after its conversion from a monomeric soluble form, which could then be used for crystallisation [54].

## Future perspectives

Despite early scepticism, and initial surprise that these complexes did survive the phase transition, MS is now firmly established in structural biology. That it is now widely accepted is evidenced by applications from other groups using the approach to complement existing structural biology techniques [55], specifically to complement EM wherein subunit stoichiometries may be ambiguous [56], to define ADP/ATP binding, and to distinguish detergent and lipids in high-resolution structures of membrane proteins with unknown density [52]. While it is not the role of MS simply to identify small molecules within high-resolution structures, it is facilitated by the developments outlined here.

Part of the future that I envisage for MS in structural biology is to move from detergent micelles to membrane mimetics [57]. To this end, new developments involving the use of nanodiscs and lipodiscs hold great promise [58]. Moving on from the many examples of prokaryotic proteins to human systems and assessing the effects of the complicated repertoire of post-translational modifications (PTMs) of eukaryotic membrane proteins on lipid interactions, while moving closer to cellular environments, are major goals for the immediate future. To this end, cross-linking approaches developed in human cell lysates [59] are particularly exciting.

A quarter of a century after my first GroEL mass spectra perhaps the aspect that I still find most exciting is the ability to form and test hypotheses based on the results of MS experiments. In this way, we have been able to propose and establish links between the changes in stoichiometry of the ribosomal stalk proteins from different species and their position on the phylogenetic tree, to extend the paradigm that intrinsic dynamics is crucial to the function of small heat shock proteins [60] and to establish roles for PTMs in bringing together protein subunits in assemblies of Hsp70 and client proteins [61].

**Figure 3.**
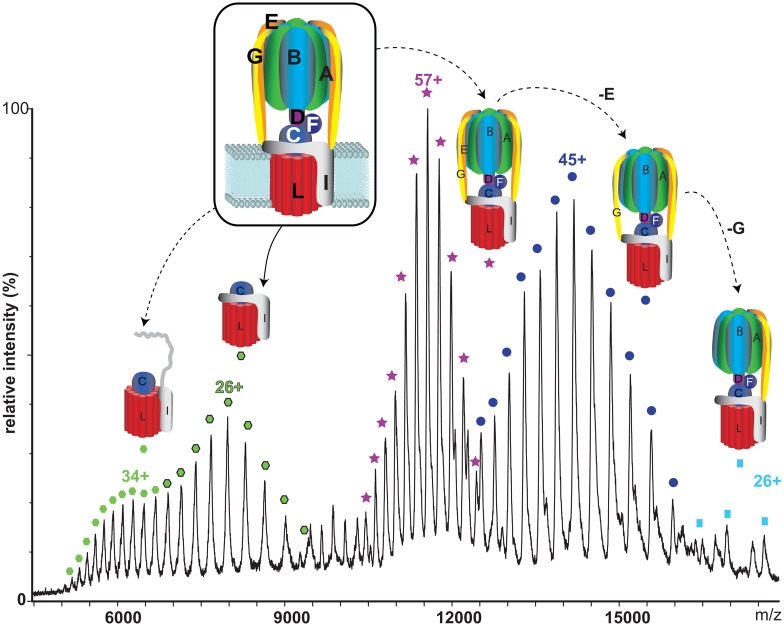
Mass spectrum of the intact rotary ATPase from *T. thermophilus* confirms the overall 29 subunit stoichiometry and enables definition of the 10 cardiolipin molecules within the rotary ring. Gas-phase dissociation products are formed at high *m*/*z* values through loss of peripheral subunits E and G.

**Figure 4.**
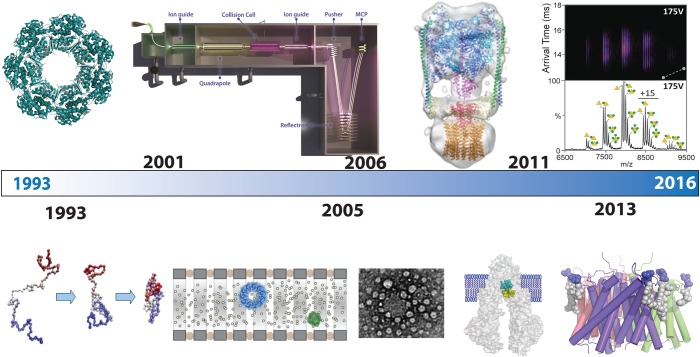
Timeline of developments from folding proteins to membrane motors. From left to right folding individual proteins (1997) and maintaining the molecular chaperone GroEL (far left) to instrumental designs for a high-mass quadrupole (top) and our first IM experiments (2005) showing separation of complexes with differing topologies but the same mass. Collection of GroEL soft-landed onto an EM grid located within the flight path of the beam. Release of membrane proteins into the gas phase led to preservation of an intact rotary ATPase (top) and fluctuations and binding of lipids and drugs to the ABC transporter P-gp as well as the first X-ray structure of the ammonia channel with lipids that stabilise the structure in the gas phase (2004).

Analogous to the role of PTMs in fine-tuning the function of soluble complexes is the influence of lipid binding on the properties of membrane proteins. The ability to rapidly test hypotheses based around questions on membrane protein lipid binding has been particularly fruitful. Specifically, we have shown that changes in lipid-binding patterns in rotary motors lead to conformational changes in the membrane ring [44]. Similarly, lipid binding was shown to have major implications for the structure and function of the ammonia channel and aquaporin Z [53] and most recently for the oligomerisation of membrane proteins (under review). By calculating the stability of all the α-helical membrane protein oligomers in the PDB, we showed that those with the lowest number of salt bridges and the weakest interface interactions were the most likely to require lipids for oligomerisation. Based on these findings, we could propose and now test the hypotheses that lipids would also mediate associations in G-protein-coupled receptors. It is this ability to respond quickly to questions with new data, spawning new hypothesis and directions, that ensures that gas-phase structural biology will remain a vibrant and exciting research activity for many years to come.

## Abbreviations

EF-G: elongation factor G
IM: ion mobility
MS: mass spectrometry
P-gp: P-glycoprotein
PTMs: post-translational modifications
sHSP: small heat shock protein
TTR: transthyretin.
